# Supply and demand in physician markets: a panel data analysis of GP services in Australia

**DOI:** 10.1007/s10754-014-9148-7

**Published:** 2014-05-14

**Authors:** Ian McRae, James R. G. Butler

**Affiliations:** 1 Australian Primary Health Care Research Institute, Australian National University, Building 63, Cnr Mills and Eggleston Roads, Acton, Canberra, ACT 0200 Australia; 2 Australian Centre for Economic Research on Health, The Australian National University, Building 62, Cnr Mills & Eggleston Roads, Acton, Canberra, ACT 0600 Australia

**Keywords:** GP markets, Panel data, Australia, Demand for physician services, Supply of physician services, 111

## Abstract

To understand the trends in any physician services market it is necessary to understand the nature of both supply and demand, but few studies have jointly examined supply and demand in these markets. This study uses aggregate panel data on general practitioner (GP) services at the Statistical Local Area level in Australia spanning eight years to estimate supply and demand equations for GP services. The structural equations of the model are estimated separately using population-weighted fixed effects panel modelling with the two stage least squares formulation of the generalised method of moments approach (GMM (2SLS)). The estimated price elasticity of demand of $-0.19$ is comparable with other studies. The direct impact of GP density on demand, while significant, proves almost immaterial in the context of near vertical supply curves. Supply changes are therefore due to shifts in the position of the curves, partly determined by a time trend. The model is validated by comparing post-panel model predictions with actual market outcomes over a period of three years and is found to provide surprisingly accurate projections over a period of significant policy change. The study confirms the need to jointly consider supply and demand in exploring the behaviour of physician services markets.

## Introduction

The operation of markets for medical care services has been an object of research in health economics for many years, and underlies decisions on health care system structures and financing. All financing decisions in health care are based, implicitly or explicitly, on underlying assumptions about the supply and demand response of the health care system. Many papers (e.g. Hadley and Reschovsky 2006; Godager et al. 2009; Baltagi et al. 2005) have particularly explored the physician supply response to changing fee structures, but few have explored joint demand and supply responses. The purpose of this work is to extend these previous studies to show that, in a general practice market with insurance rebates and flexible pricing, it is possible to model both the supply and demand responses of the market to external changes, and to explore how they interact to arrive at the final outcome of these changes. Peripherally, the study also explores the impact of GP supply on demand for services, whether this is driven by availability effects or by supplier-induced demand effects.

Ever since the appearance of Roemer’s Law over 50 years ago (Roemer 1961), the question of whether competitive markets for medical care services could achieve an optimal allocation of resources to medical care or whether the existence of supplier-induced demand would undermine competition in such markets has occupied the minds of many scholars. Numerous studies in the early empirical literature on the subject of supplier-induced demand produced evidence of a strong and statistically significant inducement effect (see e.g. Fuchs 1978; Rice 1983; Cromwell and Mitchell 1986). However, further work, including the critique by Ramsey and Wasow (1986) who had difficulty replicating results from earlier studies, cast doubts on the magnitude and significance of supplier inducement in medical care markets.

While market outcomes depend on the interaction of supply and demand, and while the existence of supplier-induced demand may affect these outcomes, the estimation of a supply and demand model is not a necessary condition for investigating the existence of supplier-induced demand. For example, Van Dijk et al. (2013) found evidence of supplier-inducement in GP-initiated contacts using a differences-in-differences approach applied to panel data that spanned a change in remuneration arrangements for GPs in the Netherlands. However, if the objective is to estimate a supply and demand model of the physician services market, then allowing for the presence of supplier-induced demand is obligatory given the controversial history of the inducement hypothesis.

The purpose of this paper is to estimate a supply and demand model for general practitioner (GP) services in Australia. While the Australian GP market is fundamentally underpinned by tax-funded national health insurance, actual fees charged are set by GPs. A substantial proportion of services (around 30 % at time of this study, around 20 % in 2012) is billed at levels in excess of the insurance rebate which means patients face an out-of-pocket cost for these services (the “balance billing” model in the United States). The main interest of the study is in understanding the overall behaviour of the GP market where both supply and demand respond to the market prices along with other factors. While the direct effect of the number of GPs on demand for GP services is incorporated in the model, whether any such effect comprises an availability effect, an inducement effect or other effects is not explored.^1^ The model is estimated using panel data for a set of moderately sized geographical regions covering the whole country for the period 1996–2003.

More recent studies of the GP market have not addressed supply and demand interactions but have addressed either supply or demand, or more frequently have addressed utilisation without attempting to separate supply and demand effects. The endeavours to separate effects have tended to follow a view that the first visit to a GP is a patient decision and later visits at least partially GP decisions. Pohlmeier and Volker (1995) introduced hurdle models to explore demand for health care following this logic, Deb and Trivedi (2002) explored finite mixture models and two part models, and Bago d’Uva (2005) explored latent class models with these authors assuming the different components of utilisation addressed patient demand and supplier inducement, but not attempting to model both supply and demand per se. Van Dijk et al. (2013) followed a similar logic making use of a natural experiment of a policy change in the Netherlands to explore moral hazard (with respect to initial visits) compared to induced demand (reflected by later visits) before and after the policy change. Where measures of access to GPs were used these were in terms of GP density, which tended to show a small positive effect of GP density on utilisation.

Other studies draw on particular data sets to address issues of supply of physician services. Baltagi et al. (2005), for example, used a dynamic labour supply approach to estimate labour supply elasticity, and Delattre and Dormont (2003) explored a physician panel with fixed fees and found that, when physician numbers increase, service volumes decrease and physicians tend to move to longer consultations with higher fees.

Studies using aggregate data generally rely on cross-section data or, where using panel data, have pooled it (e.g. Cromwell and Mitchell 1986). Leonard et al. (2009) note that all but one of the 25 studies in their systematic review of supplier-induced demand issues are cross sectional. The present study uses aggregate data, however, it differs from earlier studies using such data in that it employs panel data methods which remove the effect of any unobserved area-level heterogeneity that is stable over time, it incorporates a wide range of variables, it carefully tests for known econometric problems with the approach, and it jointly considers supply and demand. Issues raised by border crossing, a problem highlighted in the work of Dranove and Wehner (1994), are addressed directly in this study. Issues with instrument strength and validity, and issues of endogeneity, are tested vigourously to ensure potential biases are minimised or eliminated. Further, the present study, by addressing both supply and demand issues and drawing them together, facilitates the understanding of the overall market impact of policy and other external changes. Most previous studies show the impact on supply or demand only, or effects on utilisation, without addressing both supply and demand influences.

Using this methodology the contribution of this paper is twofold: it provides more reliable estimates of the determinants of GP supply and demand, and more importantly it shows the relative impact of supply and demand factors on the overall activity in this market and the fees charged.

The structure of the paper is as follows. The following section provides some background on the Australian health care system in general and markets for medical care services in particular. This provides a backdrop for the specification of the supply and demand model of GP services estimated in the study, presented in the “Model specification” section. The “Data” section provides details of the panel data set used to estimate the model, while the “Econometric methodology” section discusses the econometric methodology and other modelling issues relevant to estimating the model. The next section presents the results while the closing section of the paper places these results in the context of other Australian studies and summarises the contributions of the paper.

## The Australian health care system

Australia has a compulsory, tax-financed national health insurance scheme (Medicare). This scheme provides indemnity insurance against the cost of out-of-hospital medical services which are provided predominantly by private medical practitioners. There are no price controls on privately provided medical services in Australia. GP services are therefore charged out at a fee set by the doctor (the market price). The Medicare contribution to the cost of a GP service (the Medicare rebate) is specified in the Medicare Benefits Schedule and is a fixed subsidy set at a level the Government considers appropriate, but which does not take into account the market price of the service. The patient pays the difference between the market price and the Medicare rebate (the “gap”) as an out-of-pocket expense. If the GP accepts the Medicare rebate as full payment for the service, the rebate is paid directly to the GP and the patient makes no payment (referred to as “bulk billing”).

**Fig. 1:**
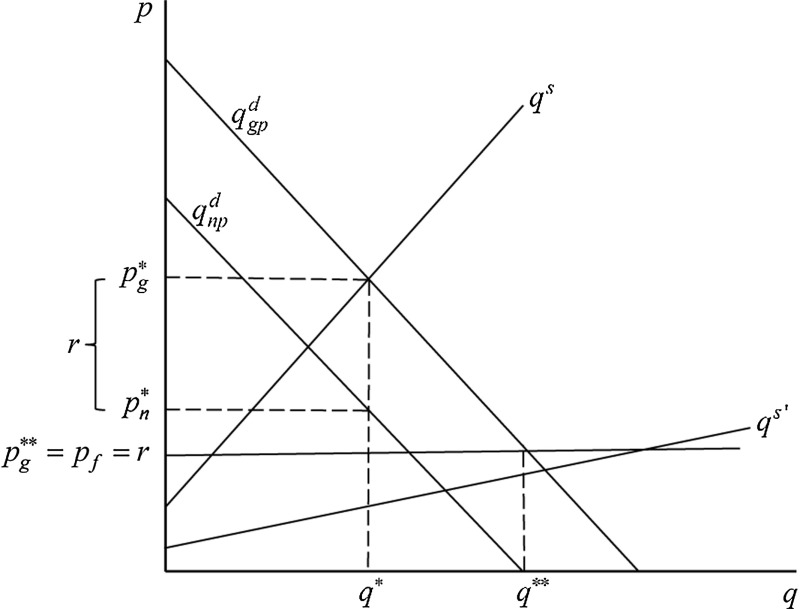
The Medicare rebate as a floor price in markets for medical services

The Medicare rebates effectively set a floor price for medical services in Australia (Butler 1994). In Fig. 1 the market for general practice services is described in a competitive market framework. The rebate $$r$$ is shown as a fixed subsidy per unit of medical service. The equilibrium market price or gross price $$p_{g}^{*}$$ and quantity traded $$q^{*}$$ are determined by the supply curve $$q^{s}$$ and the gross price demand curve $$q_{gp}^{d}$$. The vertical difference between $$q_{gp}^{d}$$ and the net price demand curve $$q_{np}^{d}$$ is the Medicare rebate. The equilibrium net price (or out-of-pocket price) borne by the patient $$p_n^{*}$$ is given by ($$p_g^{*} -r$$). If supply increases ceteris paribus then, in the absence of any direct effect of supply on demand (or supplier inducement), the market price will fall and the patient’s out-of-pocket expense will fall. If supply increases to $$q{^{s}}^{\prime }$$ in Fig. 1, the market price notionally falls to a level below the floor price $$p_g^{**}$$ but in practice, of course, falls to the floor price $$p_{f}$$ set by the Medicare rebate, and the net price to the patient is zero (the bulk-billing outcome). If the rebate is increased, $$q_{gp}^{d}$$ will shift to the right causing $$p_{g}$$ to rise and $$p_{n}$$ to fall (not shown in Fig. 1).

The analysis in Fig. 1 is predicated on a competitive market for GP services. Two previous studies of markets for GP services in Australia estimated both competitive equilibrium models and models based on monopolistic competition (Richardson 1981; Connelly 1999). Parameter estimates were similar regardless of the structure chosen. This may be because Australian GP markets are geographically quite heterogeneous, ranging from small rural towns where genuine monopolies arise to large urban areas with relatively high numbers of GPs per capita where near-perfect competition could be argued to apply, with many in-between situations of monopolistic competition or oligopoly. Based on this experience, and on the argument of McGuire (2000) that while much theoretical analysis uses monopolistic competition models most quantitative studies use competitive equilibrium models for pragmatic reasons, we have followed the equilibrium modelling approach.

## Model specification

**Fig. 2:**
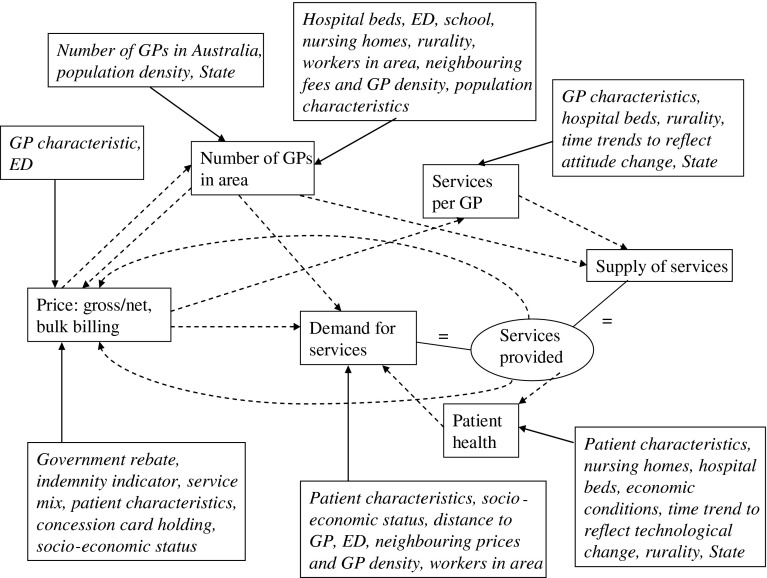
Outline of the GP model (*Standard text* endogenous variables, *italicized text* exogenous variables. *Dashed lines* indicate relationship between endogenous variables.)

The model estimated in this paper is a system of supply and demand equations for GP services that reflects the important design features of the national health insurance scheme in Australia. The general relationships between supply, demand, prices and health that are endogenous components of the model are summarised in Fig. 2. Of particular note are the following points. First, supply comprises two components: numbers of GPs, and the number of services provided per GP. This identifies two distinct processes, as government can control GP numbers to a degree but each GPs chooses the number of services he/she will provide. Second, health is treated as endogenous in the demand equation as the level of GP services provided in an area may influence the health of the area.

The model of supply and demand estimated in this paper is shown in Eqs. (1)–(7) below. The relevant sets of exogenous explanatory variables are indicated by $f_{i}$. Other variables in the schema are endogenous except the Medicare rebate ($r$) which is determined by government decision. The endogenous variable *GPd* is GP density, $h$ is health, *qGP* is quantity of services per GP, the quantity measures $q$ are expressed as services per capita and other variables are as previously defined. Error terms $\varepsilon _{i}$ are included in the stochastic Eqs. (1)– (4). Equations (5)–(7) are identities.^2^

1 $$\begin{aligned} q_{np}^d&= \alpha +\beta p_n +\gamma { GPd}+\delta h+f_1 +\varepsilon _1 \quad \ldots \end{aligned}$$

2 $$\begin{aligned} { GPd}&= \varphi +\phi p_g +f_2 +\varepsilon _2 \quad \ldots \end{aligned}$$

3 $$\begin{aligned} h&= \eta +\lambda q+f_3 +\varepsilon _3 \quad \ldots \end{aligned}$$

4 $$\begin{aligned} { qGP}&= \mu +\nu p_g +f_4 +\varepsilon _4 \quad ...\end{aligned}$$

5 $$\begin{aligned} q^{s}&= { GPd}*{ qGP}\quad \ldots \end{aligned}$$

6 $$\begin{aligned} p_n&= p_g -r\quad \ldots \end{aligned}$$

7 $$\begin{aligned} q&= q_{gp}^d =q^{s}\quad \ldots \end{aligned}$$

Following the identity in Eq. (7), the observed value of demand used in the study was the number of GP services per capita. The net fee demand curve is estimated for comparison with earlier studies, but to relate demand and supply equations a gross fee demand curve is required, and this is estimated directly.

As the purpose of this study is to relate supply and demand curves, an aggregate supply curve is required. While this can be estimated from an independently specified equation, the multiplicative structure implies a very complex equation with large numbers of interaction terms involving endogenous and exogenous variables. Estimating a simpler form of the supply equation, as done by Connelly (1999), applies severe (and very complex) constraints on Eqs. (2) and (4). The approach taken to obtaining an aggregate supply curve, therefore, is to estimate Eqs. (2) and (4) and to derive simplified supply equations at the mean points of the exogenous determinants using the identity in Eq. (5).^3^

## Data

This study uses an unpublished panel dataset comprising annual observations of private practice GP activity for 816 geographic regions for eight years based on Statistical Local Areas (SLAs) in Australia supplied by the Australian Government Department of Health and Ageing. This dataset was compiled by the Department from individual data records for the years 1996 to 2003.^4^ This dataset was combined with population census data, mortality data, hospital data and data from a range of other sources. As the analysis uses fixed effects modelling, variables which do not change over time (such as rurality/remoteness) are not relevant and are not discussed, except where used as interaction terms with other variables. A summary of the data is provided in Table 1.

**Table 1:** Mean and range of values for each variable, 816 SLAs, Selected years. Means all population weighted except GPs per SLA

Variable	Estimated mean			Min.	Max.
	1996	2000	2003	Across all years	
*GP numbers and characteristics*					
Number of GPs per SLA	26.38	26.39	26.68	0.61	329.07
Number of GPs per 1000 population (*GPd*)	1.18	1.12	1.10	0.07	6.20
Percent of GPs: female	30.42	32.66	34.59	0.00	100.00
Percent of GPs: aged under 40 years	38.29	28.77	23.13	0.00	100.00
Percent of GPs: aged 60 or more	13.85	15.18	16.34	0.00	100.00
Percent of GPs: vocationally registered or in training	82.54	85.28	87.38	0.00	100.00
Percent of GPs: trained overseas	25.40	27.21	29.60	0.00	100.00
*Quantities of services and prices* $^{\mathrm{a}}$					
Quantity of services per capita	5.52	5.28	4.73	0.34	14.08
Quantity of services per GP (*qGP*)	4,679	4,580	4,322	345	16,421
Average gross price charged ($p_{g}$)	24.45	23.33	24.51	16.39	46.58
Average net price charged ($p_{n}$)	1.67	1.98	3.21	0	25.57
Average gap when gap was charged ($p_{g}-r$)	8.52	9.09	9.81	0	89.40
Average Medicare rebate ($r$)	22.78	21.34	21.30	15.68	34.69
Service mix$^{\mathrm{b}}$ ($r/r_{s}$)	1.11	1.15	1.18	0.85	1.86
Average proportion of services bulk billed (%)	80.65	78.44	67.59	0.00	100.00
*Population and attributes*					
Population per SLA	22,434	23,467	24,358	868	191,605
Percent of population female	50.60	50.67	50.72	19.48	59.24
Percent of population aged less than 15 years	21.59	20.97	20.50	5.96	33.58
Percent of population aged 65 years or more	12.10	12.50	12.79	0.84	34.36
Percent of population with post school qualifications	16.28	18.17	19.59	2.75	50.97
Percent of population aged 15 years or more unemployed	5.51	4.63	3.97	0.00	13.79
Percent of population born outside Australia	21.94	21.72	21.56	1.58	53.47
Percent of workforce in blue-collar industries	26.15	25.58	25.15	6.28	79.73
Ratio of patients to population	0.86	0.84	0.82	0.008	2.08
*Other health facilities data*					
Hospital beds per 1,000 population	4.34	4.28	4.24	0.00	116.61
Nursing home beds per 1,000 population	3.47	3.66	3.67	0.00	37.36
*Other data*					
Crude death rate (per 10,000 population)	70.01	66.49	65.98	0.00	266.18
Percent of population with concession cards	33.93	34.66	34.94	4.34	78.22

Variable	Estimated mean			Min.	Max.
	1996	2000	2003	Across all years	
Area (square kilometres)	9,356	9,356	9,356	1.05	671,466
Population density (persons per square kilometre)	2.40	2.51	2.60	0.005	7,101.26
Number of private schools per 10,000 population	0.19	0.19	0.1919	0.00	5.49

$^\mathrm{a}$ Prices, gaps and rebates for each year have been converted to 1996 dollars by adjustment with an index based on average weekly earnings (AWE)

$^\mathrm{b}$ Service mix is measured as the ratio of the average Medicare rebate in an SLA ($r$) to the rebate for a standard item ($r_{s}$)

*Sources* A GP numbers, quantities of services and prices, patient numbers: unpublished data provided by Australian Government Department of Health and Ageing; B Population data: Australian Bureau of Statistics (2004); C Medicare rebates set by Government: Biggs (2004), Australian Government Department of Health and Ageing (2006); D Population and Attributes: (Australian Bureau of Statistics (1997, 2002, 2004); E Other Medical Services Data:; (a) Hospitals, hospital beds, emergency departments: Australian Institute of Health and Welfare (2000), APN Business Information Group (2005) and other relevant years; (b) Nursing home beds: http://www.health.gov.au/internet/wcms/publishing.nsf/Content/ageing-rescare-servlist-postcode.htm, last accessed June 2006, no longer available at this address, APN Business Information Group (2005) and other relevant years; F Other data :; (a) Crude death rates: Australian Bureau of Statistics (2005) (data available on request); (b) Concession cards: Data provided by Department of Family and Community Services, and Australian Government Department of Family and Community Services and Indigenous Affairs (2006) and other relevant years.; (c) SLA area: Australian Bureau of Statistics (2002).; (d) Population density: Australian Bureau of Statistics (2002), Australian Bureau of Statistics (2004; e) Private schools: http://www.dest.gov.au/sectors/school_education/programmes_funding/general_funding/operating_grants/general_recurrent_grants/gazettes.htm last accessed 16 October 2007, no longer available at this address, Commonwealth of Australia (2000; f) GP density by state: http://www.health.gov.au/internet/wcms/publishing.nsf/Content/health-pcd-statistics-gpnos.htm last accessed 16 October 2007, no longer available at this address

The measure of the number of GPs in each SLA is the “full-time workload equivalent headcount” (FWE-headcount). Each active GP is counted as one unit regardless of the number of services provided. A GP practising in only one SLA counts as one in that SLA. If they practise in multiple SLAs, the unit is divided according to the proportion of the Medicare schedule fees applied to the services provided in each SLA.^5^ The number of patients in each SLA is calculated using a similar method to the GP measure.

The data include the number of consultations provided by GPs, the number of those consultations bulk billed, prices charged and rebates paid, and the number of patients attending GPs.^6^ These measures are cross-classified by GPs’ age group, gender, and vocational registration status.^7^ Minor procedures and tests, which accounted for 4.3 % of services provided by GPs in 2001, are excluded. Prices and rebates are adjusted to 1996 prices by an index based on average weekly earnings (AWE).

The unpublished Medicare data on GP activity are augmented by SLA-level information from the Australian Population Censuses of 1996 and 2001 with linear interpolation and linear extrapolation where necessary. They are also augmented by an SLA-level measure of socio-economic status, the Index of Disadvantage, which is one of the Socio-economic Indexes for Areas (SEIFAs) constructed by the Australian Bureau of Statistics (2003a). SEIFA scores are ordinal ranks of socio-economic status so are classified into quintiles for the purposes of this study. SLA-level data on the numbers of hospital beds and nursing home beds are also included as these may influence the attractiveness of an area for GPs to live and work. As a measure of health, crude death rates are preferred to standardised death rates because they reflect more accurately the total quantum of ill health which both influences demand for health services and is influenced by the consumption of health services.

## Econometric methodology

### Estimation methods

The preferred approach is to estimate the model equation by equation (Greene 2003 p. 413), as this gives greater flexibility and robustness to specification error than a full system approach where specification error in any equation would affect all equations. Following Baum et al. (2002), the structural equations are estimated separately using GMM (2SLS) to accommodate the high levels of heteroskedasticity. GMM is more efficient than the standard IV estimator in this context and, given the sample sizes available, does not face the risk of possible small sample properties of GMM estimators.

To take advantage of the panel structure of this data and to benefit from the information on how the different variables change over time within each area, either fixed effects or random effects modelling are conventionally used. As a number of unobserved factors which may influence the demand equation, (e.g. perceived quality of care, use of tobacco and alcohol) are likely to be correlated with the socio-economic status variable included in the demand equation, a fixed effects (within estimator) modelling approach is preferred to random effects (Verbeek 2000). Hausman specification tests also indicate that fixed effects is preferred. SLAs are weighted by population size in estimating model parameters. Linear forms are preferred to log-log structures as levels of skewness of the major variables do not provide a strong case for transformation and the Davidson and Mackinnon PE test (Verbeek 2000) shows no preference for either form.

Following these considerations, the structural equations are estimated separately using population-weighted fixed effects panel modelling with GMM (2SLS). Modelling is undertaken within STATA using the XTIVREG2 packages (Baum et al. 2002).

### Other modelling issues

#### Identification

The order tests of identification are readily met as there are sufficient exogenous variables excluded from each equation. Rank tests are more complex with large systems and are frequently not applied when estimating these systems (Greene 2003, p. 394). Greene offers a “rule of thumb” which is met by all but the demand equation. Implementing the rank test directly for the demand equation was not possible with the non-linear constraints, so for the purposes of this test it is assumed that the system comprises only the demand Eq. (1) and (2)–(4) for the three related endogenous variables. Using the methods outlined in Wooldridge (2002), p. 220, the system meets the formal rank test for these equations.

#### Instruments

Much of the argument regarding estimation of the direct effect of GP density on demand (supplier-induced demand) using aggregate data relates to questions of identification (McGuire 2000). The variables used to identify GP density in the demand equation are the state-level GP density and the number of hospital beds per capita in the SLA. The use of state-level GP density as an instrument relies on the fact that the number of GPs in any area is determined in part by the number of GPs in Australia which is a result of government policy decisions on GP training numbers, government regulations influencing migration and registration of migrant doctors and, at least in part, variations in state-level GP density for historical reasons. The number of GPs by State uses a different definition to that used in the SLA counts, avoiding problems of additivity and enabling it to be used to instrument GP numbers by SLA. The use of the per capita number of hospital beds in an SLA as an instrument for SLA-level GP density is based on the argument that the sites of the majority of hospitals were selected many years ago, and the sites of newer hospitals depend more on availability of land than health related issues (other than a nearby population). It is therefore unlikely that there is either a causal effect from current GP density to hospital location, or any factor jointly causing GP density and hospital location.

Mortality in the demand equation is instrumented with education and country of birth (measured by the percentage of the population born outside Australia).

Price variables are endogenous in all three equations reported in this study, and are instrumented by a group of variables—the proportion of GPs who are vocationally registered, as these are more senior GPs and can broadly speaking charge higher prices, the service mix as higher prices will generally be charged for longer consultations, the level of the Medicare rebate which clearly influences the gross price but also as shown in the previous section the net price, the percentage of the population with post school qualifications which provides one proxy for available income in the community, and the number of nursing home beds per 1,000 population as those in nursing homes tend to be charged lower fees. Another possible cause of changing GP patterns of behaviour over time is concern about medical indemnity costs which began to rise markedly in 2000 (Zinn 2003). A dummy variable for the periods before and after this “medical indemnity crisis” has also been used as an instrument for prices in these equations.

As noted the equations have been estimated separately, and one of the advantages of this approach is the flexibility it permits in specification. Both theoretical reasons in the different contexts, and the formal testing of the models, has led to the inclusion of different subsets of these potential instruments in different equations.

To ensure the instruments applied in each equation have sufficient explanatory power, the thresholds identifying the weakness of instruments reported by Stock and Yogo (2005), which are based on a statistic due to Cragg and Donald (1993), are used. The final specification of all equations meets the 10 % bias criterion for the Cragg–Donald statistics (Stock and Yogo 2005). The Cragg–Donald statistics are reported for each equation.

#### Spatial correlation and border crossing

A number of recent studies using aggregate data have made adjustments for spatial correlation (Connelly and Doessel 2004). All equations estimated in this study have been tested for spatial correlation using the Moran-I statistic (Anselin 1988) and have been found not to require adjustment.

A related issue is that the total demand for GP services in any area depends on how many people travel to or from the area to obtain such services (Dranove and Wehner 1994). Direct measures of travel between SLAs are not available, but the ratio of the number of patients attending GPs in an SLA to the population of that SLA (the “border crossing ratio”) broadly reflects border crossing levels. The higher this ratio, the more likely it is that the SLA is an “inflow” area and conversely the lower the ratio. The mean value of the border crossing ratio is 0.85 as around 85 % of the population attend a GP at least once in any given year. Two dummy variables indicating SLAs falling within the lowest and highest deciles of the border crossing ratios are used to reflect travel between SLAs (low decile cutoff = 0.38, high decile threshold = 1.15).

Another way to consider border crossing is to examine neighbouring areas. Australian studies have found prices in neighbouring areas to be significant determinants of demand (Connelly 1999; Hyndman et al. 2003), while other studies found the numbers of physicians per capita in the neighbouring areas did not significantly influence demand (Fuchs 1978; Cromwell and Mitchell 1986). Both prices and GP density in neighbouring areas are therefore included in the demand Eq. (1), and are also included in the GP density Eq. (2) as GPs’ locational decisions are likely to be influenced by GP behaviour in the wider area (e.g. if higher prices may be charged in the neighbouring area, they are likely to consider working there rather than in the area under study). Neighbouring areas are however not likely to determine directly the number of hours worked by a GP once located in an area, and are therefore not included in Eq. (4). Weighted average GP prices and GP density across all neighbouring SLAs are included in this study and treated as exogenous.^8^

### Testing model validity

The models are subject to stringent testing. A version of the Ramsey RESET test adapted to the IV–GMM environment (Baum et al. 2002) is used to test for mis-specification or structural incompleteness for all models. In all cases reported here the test is met. For parsimony, actual values are not reported. Where alternative specifications are available (e.g. whether to use a continuous income measure or categorical SEIFA measures in a demand equation), Akaike Information Criteria and Bayesian Information Criteria measures are estimated and the preferred form (in this case SEIFA measures) is used. As all structural models are over-identified, the Hansen J-statistic is used to test for overall orthogonality of instruments (Baum et al. 2002). As noted in “Other modelling issues” above, instrument strength is tested and for each equation meets the 10 % bias criterion for the Cragg–Donald statistics (Stock and Yogo 2005). All equations are tested for multicollinearity by examining the Variance Inflation Factor (VIF) (Greene 2003). Where VIF values are extreme, alternate specifications are sought.

As there are clear outliers in a number of variables, alternative estimations have been undertaken with potential outliers removed. None of these estimations led to coefficients which are significantly or materially different from the values reported here.

## Results

This section presents the econometric estimates of the demand equation, the GP density equation, the quantity of services per GP equation, and overall supply offered. The resulting supply and demand curves are illustrated graphically. To simplify presentation, the results for the health equation are not presented here, but are available in a working paper (McRae 2009).

### The demand equation

Table 2 shows the parameter estimates for the estimated demand equation. The estimated net price elasticity of demand of $-0.19$ is in the conventional range (Manning et al. 1987). Cross-sectional Australian studies found net fee elasticities of $-0.22$ and $-0.31$ (Peacock and Richardson 2007; Connelly 1999). The significant elasticity on GP density of 0.129 is towards the lower end of published estimates, which vary widely between countries and estimation methods (e.g. 0.09—Cromwell and Mitchell 1986; 0.27—Carlsen and Grytten 1998; 0.46—Peacock and Richardson 2007). This suggests that the relationship between GP supply and demand in Australia (which may or may not reflect supplier inducement) may not be as large as previously estimated.

**Table 2:** Linear panel model of demand

	Coeff	Robust std. err.	Elasticity$^\mathrm{a}$
*(Endogenous variables in italics)*			
*GPs per 1,000 population*	$$0.591^{***}$$	0.126	0.129
*Average net price charged*	$$-0.461^{***}$$	0.055	$$-0.190$$
*Crude death rate*	$$0.030^{***}$$	0.006	0.392
High border crossing ratio	$$0.801^{***}$$	0.092	
Low border crossing ratio	$$-0.380^{***}$$	0.085	
Average GPs/1,000 pop’n in neighbouring SLAs	0.106	0.077	0.022
Average net price charged in neighbouring SLAs	$$0.116^{***}$$	0.038	0.049
Percentage of population female	$-0.035$	0.031	$-0.343$
Percentage of population unemployed	$$0.081^{***}$$	0.021	0.074
Percentage of population aged 65 or over	0.002	0.029	0.004
Percentage of population aged under 15	$-0.037^{*}$	0.022	$-0.149$
Percentage of population Indigenous Australians	$-0.027$	0.024	$-0.011$
Indicator of second SEIFA quintile	0.013	0.044	
Indicator of third SEIFA quintile	0.079	0.065	
Indicator of fourth SEIFA quintile	0.027	0.089	
Indicator of fifth SEIFA quintile	0.138	0.118	

Exogenous variables used to instrument the endogenous variables: Percentage of GPs vocationally registered; Medicare rebate for a standard consultation; Indicator of the indemnity crisis; Number of GPs by State; Number of hospital beds per capita; Percentage of population with post school qualifications; Percentage of population born overseas

Hansen J statistic: *p* value=0.49; Cragg–Donald statistic = 21.58

$${*}$$ Significant at 10 %

$${**}$$ Significant at 5 %

$${***}$$ Significant at 1 %

$^\mathrm{a}$ Elasticity at 8 year mean values, not relevant for indicator variables

Mortality dominates the effects of personal characteristics other than unemployment. Indicators of high and low levels of border crossing proved to be significant. As in previous studies, the coefficient on GP density in neighbouring SLAs is insignificant but those on net prices in neighbouring SLAs are significant and positive but with very small elasticities.

### The GP density equation

Table 3 shows the parameter estimates for the estimated GP density equation. The supply of GPs in any SLA depends on the number of GPs active in the country as a whole, and their choice of location. As expected, to a substantial degree, local GP densities follow overall patterns with the GP density in neighbouring SLAs exerting a positive and significant effect. State-level GP density has a significant positive effect with a coefficient near unity in the GP supply equation. The gross price coefficient is significant and positive with an elasticity of 0.69, suggesting potential income is a determinant of GP locational choices. The coefficient on the indicator of high border crossing ratio is also positive and significant, implying that GPs are drawn to areas like central business districts which provide access to patients.

**Table 3:** Linear panel model of GP density

	Coeff.	Robust std. err.	Elasticity$^{\mathrm{a}}$
*(Endogenous variables in italics)*			
*Average gross price charged*	$$0.033^{***}$$	0.005	0.690
High border crossing ratio	$$0.230^{***}$$	0.028	
Low border crossing ratio	$$-0.147^{***}$$	0.024	
Average GPs/1,000 pop’n in neighbouring SLAs	$$0.210^{***}$$	0.029	0.197
Average gross price charged in neighbouring SLAs	$$-0.024^{***}$$	0.005	$$-0.499$$
Percentage of population aged under 15	0.003	0.005	0.054
Percentage of population aged 65 or over	$$0.052^{***}$$	0.006	0.571
Percentage of employed people who are blue collar workers	$$-0.008^{***}$$	0.003	$$-0.191$$
Hospital beds per 1,000 population	$$0.005^{***}$$	0.002	0.019
Private schools per 1,000 population	0.444	0.461	0.008
Nursing home beds per 1,000 population	0.001	0.004	0.003
Number of GPs per 1,000 pop’n in the state	$$0.952^{***}$$	0.083	1.070

Exogenous variables used to instrument the endogenous variable: service mix; Medicare rebate for a standard consultation

Hansen J statistic: *p* value = 0.59; Cragg–Donald statistic = 889.56

$${*}$$ Significant at 10 %

$${**}$$ Significant at 5 %

$${***}$$ Significant at 1 %

$^\mathrm{a}$ Elasticity at eight year mean values, not relevant for indicator variables

The positive coefficient on GP density in neighbouring areas suggests that there are broad areas which are attractive to doctors. GPs are also attracted to areas where the neighbouring SLAs have relatively low fees, meaning the areas they choose have relatively high fees.

### The quantity of services per GP equation

Table 4 shows the parameter estimates for the estimated quantity of services per GP equation. The quantity of services offered per GP depends on gross prices, the characteristics of the GP and, to a lesser degree, the characteristics of the area.

**Table 4:** Linear panel model of services per GP

	Coeff.	Robust std. err.	Elasticity$^{\mathbf{a}}$
*(Endogenous variables in italics)*			
*Average gross price charged*	$$-0.052^{***}$$	0.007	$$-0.270$$
Percentage of GPs female	$$-0.013^{***}$$	0.001	$$-0.095$$
Percentage of GPs aged 60 or over	$$-0.007^{***}$$	0.001	$$-0.024$$
Percentage of GPs aged under 40	$$-0.009^{***}$$	0.001	$$-0.062$$
Percentage of GPs vocationally registered	$$0.023^{***}$$	0.001	0.425
Hospital beds per 1,000 population	$-0.003$	0.002	$-0.003$
Time trend	$$-0.063^{***}$$	0.004	$$-0.063$$
Time trend/inner regional interaction	$$-0.031^{***}$$	0.005	$$-0.006$$
Time trend/rural interaction	$$-0.022^{***}$$	0.007	$$-0.003$$

Exogenous variables used to instrument the endogenous variable: Service mix; Medicare rebate for a standard consultation; Percentage of population with post school qualifications; Nursing home beds per 1,000 population

Hansen J statistic: *p* value = 0.793; Cragg-Donald statistic = 1049.11

$$^{*}$$ Significant at 10 %;

$$^{**}$$ Significant at 5 %;

$${***}$$ Significant at 1 %

$^\mathrm{a}$ Elasticity at eight year mean values, not relevant for indicator variables

The significant negative response of GP activity levels to gross prices shown in Table 4 is unexpected, but is stable across a range of specifications including those with price-squared terms. Published estimates on the price elasticity of supply of physician services range from elasticities of $-0.2$ (Brown 1994) to 0.55 (Baltagi et al. 2005). An Australian survey of GPs (Access Economics Pty Ltd 2002) found the supply curve for GPs to be backward-bending at a fee equivalent to roughly twice the then hourly wage rate, giving a curve which was upward sloping for most GPs. Connelly and Butler (2012) also report evidence suggesting a backward bending cupply curve of GP services in Australia based on time series analyses of the effects of major changes in the Medicare rebates for such services in the mid-2000s.

While negative, the impact of prices on services per GP was small after allowing for GP characteristics and time trend effects, as the average real gross price increased only 0.15 % from 1996 to 2003, and the effect of shifting between the 10th and 90th percentile of price is only 8% of the average services per GP. While this suggests there may be income effects, with GPs who are in areas where they can charge higher prices taking the opportunity to work fewer hours, such effects are small. Shifts in the position of the curve, substantively due to the time trends, rather than movement along the curve are the main determinants of services offered per GP.

### Overall supply offered

At the mean of all exogenous variables across the eight years, the relationships between GP density, the quantity of services per GP and the gross price are:^9^

8 $$\begin{aligned} { GPd}&= 0.666+0.0195p_g \quad \ldots \end{aligned}$$

9 $$\begin{aligned} { qGP}&= 5.822-0.0517p_g \quad \ldots \end{aligned}$$

The overall formulation is obtained as the product of these two equations (bootstrapped standard errors in parentheses):10This backward-bending supply curve turns at a fee of $39.03 (1996 prices), well above the 99th percentile of the eight year mean gross price of $31.10. The gross price elasticity of supply at the mean gross price across the eight year period is 0.14 (SE 0.09) which is not significantly different from zero.

### Supply and demand curves

**Fig. 3:**
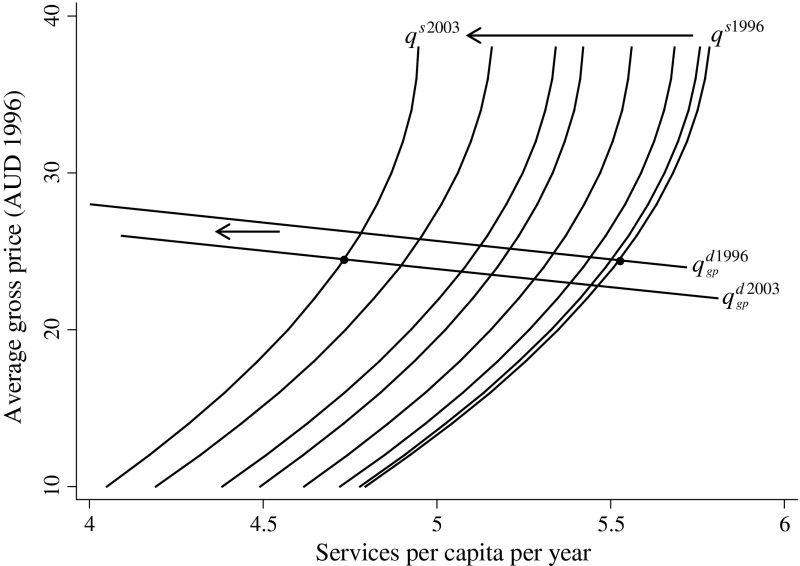
Estimated annual supply curves for 1996–2003 and demand curves for 1996 and 2003 (average gross prices in Australian dollars, 1996 prices)

Figure 3 illustrates supply curves for each year for the time period covered by the study (1996–2003). As the fixed effects model assumes coefficients do not change over time, the slopes in the GP density and the services per GP curves apply each year. Using the relevant values for each year, overall relationships between price and supply are derived, giving a series of backward-bending supply curves which are upward sloping across most of the plausible average gross fee range, and which shift to the left over time. The shift is due to the combination of changes in the overall GP density and the trend in services per GP.

The estimated demand curves for the first (1996) and last (2003) years of the series are also shown in Fig. 3. Net price demand curves shift very little due to the variables shown in Table 1, but when the curves are plotted against gross prices (as in Fig. 3) they shift to the left as the Medicare rebate fell by 12.1 % in real terms between 1996 and 2003 (using the AWE price adjustment).

The overall effect of the demand curve and the supply curve shifting left is that the real gross price does not rise and volumes fall. The stable gross price together with a falling real Medicare rebate means increasing net prices. As supply is highly inelastic, the change in volume is mainly caused by the shift in the supply curve. The effect of the shift in the demand curve on volume of services is trivial with no change in supply.

### Validation of the supply and demand relationship

After 2003, the last year of the panel dataset on which the model is based, the Australian government made a series of policy changes regarding general practice. These changes provide an opportunity to use the estimated supply and demand equations to predict outcomes over the period 2003–2006. As the overall demand and supply curves shown in Fig. 3 are set at the mean values of all independent variables (apart from price) for the relevant year, the curves for 2006 were derived using the mean values for these variables at 2006 (with trend variables simply moved through time). In the case of the demand curve this was done directly. In the case of the supply curve the GP density and services per GP equations were estimated at the mean and again multiplied as for Eqs. (8), (9) and (10) in this paper.

The policy changes after 2003 significantly increased Medicare rebates for GP services. In addition, financial incentives were offered for services provided to concession card holders, and to children aged under 16 years, when they were bulk billed. Also, the general practice workforce was increased with GPs from overseas and locally trained GPs. Including the new incentive payments as a notional part of the rebate, between 2003 and 2006 the average increase in the real value of rebates per service was 17 %. Average GP density is estimated to have increased by 1.0 % over the three years to 2006.^10^

These changes provide an opportunity to use the estimated supply and demand equations to predict outcomes over the period 2003–2006. The projected supply and demand curves for 2006 meet at a point which under-estimates the change in prices by $0.30 per service, and over-estimates GP service utilisation by 0.1 services per capita. This is much closer to the actual outcome than expected, given the “shock” of the major increase in rebates together with the introduction of the bulk billing incentives.

## Discussion and conclusion

There have been many analyses of GP issues in Australia (Young et al. 2001; Khan et al. 2004; Savage and Jones 2004) as well as the many international studies ranging from Manning et al. (1987) based on the Rand health insurance experiment to the more recent latent class analyses (e.g. Bago d’Uva 2005) and opportunistic uses of natural experiments (e.g. Van Dijk et al. 2013). However, only the work of Richardson (1981); Peacock and Richardson (2007) and of Connelly (1999) attempted to address the full range of relationships within the GP market.

Both Richardson and colleagues and Connelly found price elasticities of demand in the conventional range. Our estimate of price elasticity of demand ($-0.19$) is lower than the previous Australian estimates, probably due to the use panel data estimation techniques which remove many of the missing variable biases. Our estimates are, however, of the same order as most other studies that have appeared since Manning et al. (1987).

The estimated elasticity for GP density on demand (0.129) is considerably less than those of Richardson and colleagues and significantly different to the Connelly estimate. They are also somewhat less than the estimates of Mitchell and Cromwell (1995) who also addressed the impact of the Medicare reforms, but focused on particular service types. This is probably due to the explicit allowance for border crossing effects which have been argued to have a major impact on estimated effects of GP numbers on demand (Dranove and Wehner 1994). Our estimate is of the same order as Cromwell and Mitchell (1986) and of Escarce (1993) (who used reforms to the American Medicare fee structure to generate a natural behavioural experiment).

The price elasticity of supply of GP services estimated in this paper (0.14) is small and not statistically significantly different from zero, suggesting the supply curve is near vertical. While Brown (1994) and Connelly (1999) both found negative price elasticities of supply, their estimates were also not statistically significant.

With a near vertical supply curve, shifts in the demand curve due to changing GP density have very little effect on price or volume of services, and the main driver of volume is the position of that supply curve. The relatively low elasticity of GP density on demand combined with a near vertical supply curve therefore mean that changes in GP density have very little effect on equilibrium market outcomes.

This study has a number of potential limitations. Being an ecological study, it faces the risk of the ecological fallacy. While this cannot be ignored, the broad similarity of results, e.g. on the price elasticity of demand, across the range of methods in the literature (including individual, ecological, natural experiments) gives provides confidence in the results. Regarding the use of instrumental variables, there will always be debate about the suitablilty of instruments but the instruments used here are logical and are formally tested for all the conventional concerns. Finally, as GP density is used as the measure of competition, it is not possible to separate availability effects of access to GPs from any supplier-induced demand effects. The separation of these effects must rely on evidence from natural experiments such as that addressed by Van Dijk et al. (2013), or from hurdle-type models such as those of Pohlmeier and Volker (1995).This study extends previous work in this area in several directions. GMM estimation with aggregated panel data meets the relevant econometric tests and provides a plausible description of the system of relationships within the GP market in Australia. Use of panel data analysis techniques, a wide range of explanatory variables, and some direct allowance for border crossing addresses previous methodological concerns with aggregate data (McGuire 2000). A two stage approach of estimating, then combining, the GP density equation and quantity of services per GP equation generates a plausible overall supply equation which provides relatively accurate predictions.

The jointly estimated supply and demand model developed in this paper provides an enhanced capability to analyse the effects of policy reforms and a pragmatic approach to predict policy outcomes in markets for GP services characterised by a fee for service structure and flexible pricing. An example of this is a practical application of the model involving estimation of the potential effect of the Global Financial Crisis (GFC) on GP markets by McRae and Paolucci (2009).

This study has successfully applied panel data analysis of aggregate data to develop estimates of supply and demand in the Australian GP market. The approach is applicable in any other similar environment, and emphasises the need to understand both the supply and demand structures in considering policy changes in healthcare markets. The model allows the estimation of market outcomes based on both the supply and demand equations, and makes clear that any projections for policy or other purposes in an uncapped fee-for-service environment such as the Australian GP market must explicitly consider both supply and demand effects.

## Footnotes

1 For an extended discussion of these effects, see McGuire (2000).

2 Note that the net price and gross price equations are fundamentally identical apart from small shifts in the rebate due to different mixtures of long and short consultations. The net price equation is reported as this provides more meaningful elasticities.

3 Note that GP density is not included as a determinant of the number of services per GP in Eq. (4). While the data used to estimate the model show a strong correlation between GP density and services per GP, this is more arithmetical than behavioural: the majority of areas have services per capita in a relatively narrow band, so GPs per capita and services per GP are automatically negatively correlated. The number of services each GP chooses to provide is expected to depend more on their individual characteristics (gender, family situation, age etc) than on the density of GPs in the area.

4 Further information on the SLA classification can be found in Australian Bureau of Statistics (2003b).

5 GPs providing services less than $1,000 in value (based on Medicare schedule fees) per year were excluded from the dataset when it was compiled by the Department of Health and Ageing.

6 Prices charged will be influenced by consultation length. As direct measures of consultation length are not available, “a proxy ‘service mix’ is used.” The definition of this proxy is provided in Table 1.

7 Vocational registration generally means fellowship of the Royal Australian College of General Practitioners and completion of a general practice training program.

8 It can be argued that price and the number of GPs in an SLA influence all neighbouring areas so price and GP density in those areas should not be assumed to be exogenous. However, the assumption of exogeneity is justified for two reasons. First, prior evidence and the evidence here is that price and GP density in an SLA have only a small effect on demand in a neighbouring SLA relative to the effect of price in that neighbouring SLA. Second, any reverse causality is further reduced by including all surrounding SLAs in the average for neighbouring variables. Formal testing of endogeneity in the relevant equations supports this view.

9 In this model the gross fee comprises the exogenous government rebate plus the market-determined net fee. In considering overall rather than local effects, given that the gross fees move in part with the government rebate, the coefficient on the gross fee in Eq. (8) was re-estimated without the effect of the gross fee in the neighbouring areas.

10 Population estimates from Australian Bureau of Statistics (2006). GP data from Medicare Australia http://www.medicareaustralia.gov.au/statistics/imd/forms/gpStatistics.shtml (last Accessed 16 Oct 2007).
